# Aesthetic Literacy in Young People’s and Adults’ Awareness From a Developmental Learning Perspective

**DOI:** 10.3389/fpsyg.2021.638920

**Published:** 2021-05-21

**Authors:** Gustavo Cunha de Araújo, José Carlos Miguel, Rosane Gomes de Araújo

**Affiliations:** ^1^Department of Rural Education, Universidade Federal do Tocantins (UFT), Tocantinópolis, Brazil; ^2^Didactics Department, São Paulo State University (UNESP), Marília, Brazil

**Keywords:** aesthetic literacy, developmental learning, study activity, rural education, comic books

## Abstract

In Brazilian schools, many teachers do not organize their teaching and students’ tasks and actions in a way that facilitates theoretical thinking based on the abstraction and generalization of the work content. Because many students struggle to accomplish the tasks and actions themselves, teachers guide them. Over time, the students begin to have more autonomy in executing the proposed activities, as they completed mental operations while learning. This article aims to investigate how young people’s and adults’ awareness of the countryside is formed based on visual elements and writing, facilitating an understanding of their reality. A didactic–formative experiment was performed based on the cultural–historical theory. The comic books produced by the participants allowed them to develop their overall thinking, moving from the abstract to the concrete. They also formed an awareness of reality, which allowed them to have greater autonomy in the production of these stories as a means of representation and transformation of reality.

## Introduction

Rural education has been struggling to assume an important role in Brazilian higher education by attempting to articulate popular ideas using scientific knowledge and creating new forms of research, which is important in thinking about and proposing new public policies that contribute to the advancement of rural education in Brazilian society.

However, there is a lack of research related to the literacy of young people and adults in field education, especially regarding reading and writing about art. In view of this, the results of this study can help expand new research on the subject by contributing to the advancement of knowledge production in other international contexts.

According to data from the last Brazilian Census of 2010, the state of Tocantins has a significant percentage (21.19%) of people living in the countryside, compared to the Brazilian average (15.65%). However, a large number of people who live in the countryside have low levels of schooling and experience great social, economic, and political inequalities.

This article aims to investigate how young people’s and adults’ awareness of the countryside is formed based on visual elements and writing in order to understand their reality. This study was based on the cultural–historical theory and used a didactic–formative experiment developed in a History in Comic Books course for the degree in rural education with a qualification in Art and Music of the UFT, Tocantinópolis, Brazil.

We hypothesized that comics, as a broader form of literacy and consisting of written (verbal text) and visual (drawings) elements, contributes to young people’s and adults’ awareness of rural education. For the assessment, we used a study activity developed from the perspective of developmental learning. In fact, the aesthetic^1^ literacy that we propose in this study can help this type of student, or even an illiterate person, understand a text consisting of only letters and words that would otherwise be difficult for them to comprehend.

## Materials and Methods

### Measures and Procedures

Using a study activity^2^, we developed an experiment with tasks and actions that were performed in 10 experimental classes by young and adult students of the rural education program of the UFT based on texts and drawings they produced manually. That is, we executed the experiment (Tables 1, 2) in 10 History in Comic Books classes over almost 8 months. The main idea of a didactic–formative experiment is that the teaching organized by the study activity would increase the quality of learning and, consequently, the full development of students, according to Davídov (1978, 1988, 1997), Vygotsky (1998, 1999, 2000, 2001, 2007, 2009, 2010), and Aquino (2013, 2015, 2017).

**TABLE 1:** Experimental didactic system synthesis.

**Didactic–formative experiment**		
1st Stage	Theoretical research and diagnosis of the studied reality (rural education in Tocantins, Brazil)	We conducted a bibliographic review based on the cultural–historical theory, which allowed us to understand the essence of the researched reality
2nd Stage	Elaboration of the didactic–formative experiment	The experiment was based on the teaching plan for the subject “History in Comic Books” with the objective of proposing changes in the work content based on the theory of developmental learning
3rd Stage	Development of the didactic–formative experiment	The experiment was developed in 10 classes on the subject “History in Comic Books,” with study tasks that were carried out by young people and adults from rural education
4th Stage	Categorization, discussion, and analysis of the data generated in the experiment	The data were produced in the experimental classes (comics produced by them) and analyzed using the cultural-historical theory

*Source: Elaborated by the authors, based on Aquino (2013, 2015, 2017).*

**TABLE 2:** Experimental didactic system.

**UNIVERSIDADE FEDERAL DO TOCANTINS – UFT COURSE FOR THE DEGREE IN RURAL EDUCATION WITH A QUALIFICATION IN ART AND MUSIC CAMPUS TOCANTINÓPOLIS, BRAZIL TEACHING UNIT PLAN – HISTORY IN COMIC BOOKS**				
Main Objective: To investigate how aesthetic literacy is developed through visual signs and writing to understand the reality of young people and adults in rural education				

**1ST STUDY TASK: CHARACTERIZATION OF THE COMIC BOOKS**				

**Objective**	**Content**	**Methodological development**	**Evaluation**	**Didactic materials**

-Identify the characteristics of comic books	-Comic Books concept, history, and characterization	1st action: Presentation of the Subject Plan and the theme to be studied	-Observation of the discourse produced by students regarding comics	Texts, image, Projector (Datashow)
		2nd action: Next, show different pictures and ask the students if a sequence of pictures can be considered comics? And whether drawings with verbal texts are also comics?		
		3rd action: Based on the students’ answers, ask: what are comic books? When did comics come about? Ask the students if they have ever read a comic book; if they are familiar with any character, story, artist; if they like comics; if they read them in school?		
		4th action: What relationship do you have with reading and writing?		
		5th action: In order to broaden the understanding of comic books and their characterization, we asked: How are the characters’ lines represented and developed in the comics? What about the sounds in the comics? What are the other elements of the visual language of comics?		

**2ND STUDY TASK: THE READING OF VERBAL AND NON-VERBAL TEXT IN COMIC BOOKS**				

**Objective**	**Content**	**Methodological development**	**Evaluation**	**Didactic Materials**

-Understand what is a verbal text and a non-verbal (visual) text	-Characterization and comprehension of a verbal text and non-verbal (visual) text	1st action: Review with the students what they learned in the 1st lesson. Then ask the about their understanding of the text; how a text is constituted; if a text can be visual (image), if it can have different meanings. What is a written text	-Observation of the discourse produced by students regarding comics, reading and writing	-Projector (Datashow)
		2nd action: After the students’ answers, present them comic books with only images and comic books with images and text. Then ask the students if they can differentiate verbal text from non-verbal text; if they can produce verbal and non-verbal stories		-Comic Books
		3rd action: What do students understand by language?		
		4th action: Next, ask the students about their understanding of reading. What is reading? Does reading occur only through writing? Or by images as well?		
		5th action: Next, ask the students what they understand by writing		

**3RD STUDY TASK: READING AND COMPREHENSION OF COMIC BOOKS**				

**Objective**	**Content**	**Methodological development**	**Evaluation**	**Didactic materials**

-Reading comic books in the classroom	-Characterization and comprehension of comic books from reading a comic book	1st action: To bring comic books in the classroom for the students and ask them to read the comics	-Observation of the discourse produced by students regarding comics, reading and writing	-Projector (Datashow)
-Develop reading skills		2nd action: Next, present some pictures of comics and ask the students if these stories are forms of reading and writing		-Comic Books
-Know the elements of the visual language of comic books				

**4TH STUDY TASK: PRODUÇÃO DE TEXTOS VERBAIS NAS HQS**				

**Objective**	**Content**	**Methodological Development**	**Evaluation**	**Didactic Materials**

-Produce verbal texts from comics books	-Text production	1st action: Review what the students learned in the 3rd lesson	-Observation of the discourse produced by students regarding comics, reading and writing.	-Datashow
	-Narrative text			-Sheets of paper for the production of the stories
-Understand what a narrative text is	-Life stories and imagination	2nd action: Based on their experiences with reading, and the last lessons, ask the students to create a narrative text (text that tells a story) about the comic they will produce. The text should have characters, elements of the visual language of comics, scenarios, and other elements		
		3rd action: Give the students sheets of paper to write the text of the stories		

**5TH STUDY TASK: NON-VERBAL TEXT PRODUCTION (VISUAL) IN COMIC BOOKS**				

**Objective**	**Content**	**Methodological development**	**Evaluation**	**Didactic materials**

-Initiate students into visual art practices	-Visual production of a comic book	1st action: Review what they saw in the 4th lesson. Ask the students how a drawing can be made. What materials can I use to make a drawing?	-Consistency in the drawings made with the texts produced	-Sheets of paper
-Produce non-verbal text (drawings) from the stories		2nd action: Introduce students to a geometric drawing technique, sketching different figures for the production of the comic books drawings. Is it possible to draw different objects with this technique?		-Pencils
		3rd action: Give the students the materials for the visual production of the comics: sheets of paper for sketching, pencils, erasers, rulers		-Eraser
		4th action: Based on the text created by the students, ask them to create the drawings of the stories		-Rulers

**6TH STUDY TASK: NON-VERBAL TEXT PRODUCTION (VISUAL) IN COMIC BOOKS - CONTINUATION**				

**Objective**	**Content**	**Methodological development**	**Evaluation**	**Didactic materials**

-Produce non-verbal text (drawings) from the stories	-Visual production of comic books	1st action: Ask the students to continue creating the drawings of the stories	-Consistency in the drawings made with the texts produced	-Sheets of paper
	-Visual references to objects and different elements of everyday life	2nd action: Introduce students to different objects and elements of everyday life, so that they can expand their visual references in the development of the comics		-Pencils
-Create the drawings for the comic books				-Eraser
-Extend visual memory				-Rulers

**7TH STUDY TASK: NON-VERBAL TEXT PRODUCTION (VISUAL) IN COMIC BOOKS - CONTINUATION**				

**Objective**	**Content**	**Methodological development**	**Evaluation**	**Didactic materials**

-Produce non-verbal text (drawings) from the stories	-Final art	1st action: Ask the students to continue creating the drawings of the stories	-Consistency in the drawings made with the texts produced	-Sheets of paper
				-Pencils
				-Eraser
-Start the final art for the comic books		2nd action: Give the students other sheets of paper. Ask the students to start the final artwork of the stories produced		-Rulers
				-Colored pencils
				-Black pen

**8TH STUDY TASK: NON-VERBAL TEXT PRODUCTION (VISUAL) IN COMIC BOOKS - CONTINUATION**				

**Objective**	**Content**	**Methodological development**	**Evaluation**	**Didactic materials**

-Produce non-verbal text (drawings) from the stories	-Final art	1st action: Ask the students to continue creating the drawings of the stories	-Consistency in the drawings made with the texts produced	-Sheets of paper
				-Pencils
				-Eraser
				-Rulers
				-Colored pencils
				-Black pen

**9TH STUDY TASK: PRESENTATION AND ANALYSIS OF THE COMIC BOOKS PRODUCED**				

**Objective**	**Content**	**Methodological development**	**Evaluation**	**Didactic materials**

-Analyze the comic books produced	-Comic Books; reading comprehension and writing	1st action: Ask the students what their impressions were of the comic books produced during the lessons, and the difficulties and challenges they faced, among others	-Student participation in the analysis of the comic books and the development of autonomy and self-control	-Comic Books
-Promote student autonomy and self-control				
		2nd action: Analyze the comic books produced by the students, asking each student to present his or her story		

**10TH STUDY TASK: COMIC BOOKS EXHIBITION**				

**Objective**	**Content**	**Methodological Development**	**Evaluation**	**Didactic Materials**

-Present the comic books produced by the students	-Exhibition of the comic books produced	1st action: Ask the students to assemble and present their comic books in the university lobby. Ask each student to explain his or her comic to everyone	-Interaction of students with colleagues and the academic community	-Comic Books
-To promote the student’s cultural and artistic knowledge				
-Extend their reading and writing development				

*Source: Elaborated by the authors.*

Davídov (1988, 1978) emphasizes that this method is based on the structure and procedures of new teaching programs. The teacher’s mediation is fundamental, through procedures that aim to activate other levels of development in the students, which makes it possible to boost learning. It starts from the abstract and progresses to the concrete, rather than the opposite, because it should start from what the person already knows.

Students used words as an instrument to communicate, and developed in the experiment, and consequently in the activity (tasks and actions), the beginning of the formation of concepts. Based on this, it is possible to consider that studies on concept formation are materialized by a didactic–formative experiment. Thus, the experiment briefly followed the four steps described below (Table 1).

We reviewed the didactic–formative experiment using comics as the core of the study activity conducted. By using art to elaborate the experiment, we tried to show that through art and the creativity developed by peasant youth and adults, it is possible to transform the reality in which they live and produce knowledge based on it.

From our point of view, the didactic–formative experiment helped us organize the teaching by enabling the young students and adults to develop their psychic functions during the formation of concepts and, consequently, in the construction of their integral development, considering learning as a factor in this development.

We present the details of all the steps of the experiment developed (Table 2) with young people and adults in rural education, as well as the study tasks and actions conducted with these students. The activity the student performed was reading and production of a comic book that contained verbal texts and drawings. In this research, we seek to understand comics as a study activity, which can help develop the superior psychological functions of young and adult peasants through the text and drawings they contain.

In this study activity, students improve themselves through reflections on the ongoing learning, thus driving development. For Davídov et al. (2014, p. 102), this type of activity provides students the opportunity to overcome their own limitations in tasks, both privately and in relationships with other people, i.e., “to teach and change themselves, the person must first, know about the limitations and, second, be able to transform the limits of their abilities.”

Thus, the subject of History in Comic Books was planned according to the historical and social contents to be taught. The teacher sought to identify the conceptual core of the content taught, considering the description of a conceptual core that allows the generalization of the content to be formed, the placement of the problem that will be researched in learning, presentation of the content in relation to the concepts worked, preparation of objectives and study tasks, and evaluation to be performed.

This study was approved by the Brazilian Ethics and Research Committee no. 59558116.6.0000.5406 and is linked to São Paulo State University and Universidade Federal do Tocantins, Brazil.

### Participants

The research participants included 36 young people and adults from the rural education course with arts and music qualifications from the Universidade Federal do Tocantins, Tocantinópolis, who were enrolled in a course titled History in Comic Books. The participants (between 25 and 50 years old) signed a free and informed consent term and voluntarily participated in this research. Of these, 29 were women, and 7 were men. The students were identified by codes (A1, A2, etc.) to preserve their anonymity and to meet the ethical principles for conducting research using human subjects.

### Data Analysis

The studies of Vygotsky and his collaborators using the cultural–historical theory, based on historical and dialectic materialism, substantiated the analyses we carried out. Using this form of analysis and interpretation of the data, from the perspective of cultural–historical theory, meant not only understanding history in the process of change of an individual—as a historical subject and the contradictions present in reality (Marx and Engels, 1974)—but also understanding the development of mental processes based on a study activity.

Therefore, it is necessary to understand the relationship between a person (young people and adults) and nature in its historicity and transformation. That is, the reality and the object of this study are not understood by the researchers as immutable or neutral but instead understood in terms of constant movement and change; therefore, it is necessary to understand their totality through abstraction (theoretical thought).

## Results

The texts produced by the students covered diverse themes. Of the 40 students enrolled in the course, 39 elaborated these texts. Of these, 18 are imaginative stories, 14 are about daily life/experiences, four are fantasy stories of the imagination, three represent real/true events, and one deals with religion. However, for this article, we have included the verbal text of a story produced by one student and a drawing of a comic created by another student, both participants in this research, and analyzed them in light of the cultural–historical theory. It is important to emphasize that both stories were produced manually by the students during the experiment without aid or technological resources and guided only by a history in comic books professor.

One aspect from the countryside highlighted in one of the stories produced here shows how this space is permeated with fights and disputes. The stories produced by the students made this clear. For example, student A5’s story titled “Destruction of the Forest” describes how agribusiness still influences Brazil’s agricultural and educational policy:

Title Text History: “Destruction of the Forest”

Characters of the student’s history: Laura and Francisco:

History:

Laura – “Aaaaaah!”

Laura – “But what is this Francisco! What’s happening here?”

Francisco – “Laura, it is a tractor cutting down the forest for soy planting!”

Tractor – “Blah, blah, blah!”

Francisco – “Laura, this is the result of the agricultural policy adopted by our government!’

Laura – “It can’t be Francisco, how can such a beautiful forest be destroyed for soy planting!”

Laura – “My God!”

Francisco – “Laura is how farmers linked to agribusiness and big capital do it.”

Francisco – “They destroy the whole forest without mercy!”

Laura – “Francisco, what is going to happen now to the river that rises inside the forest? The one that is being destroyed and that passes through our village?’

Francisco – “These farmers only care about bigger profits every time. That is why they do not respect the environment.”

Francisco – “That’s why Laura, two things can happen with the river: it will dry up or silt up because of the deforestation!”

Laura – “Do you mean Francisco that we won’t be able to bathe in the river anymore?”

Francisco – “No, because they plant poison in the soil! And when it rains, the rain water carries the residues of the poisons into the River, Laura!”

Francisco – “These farmers only care about their profits, and that’s why they don’t respect the environment, do you understand?”

Laura – “In this sense, they are practicing unsustainable agriculture, because they destroy the nature, the soil, the waters, poisoning the rivers and eliminating all the forms of life! It’s not really sustainable, Francisco!”

Francisco – “Besides Laura, the seeds they plant are transgenic, that is, they are “invented” in laboratories. They give produce only if they use poisons.”

**FIGURE 1:**
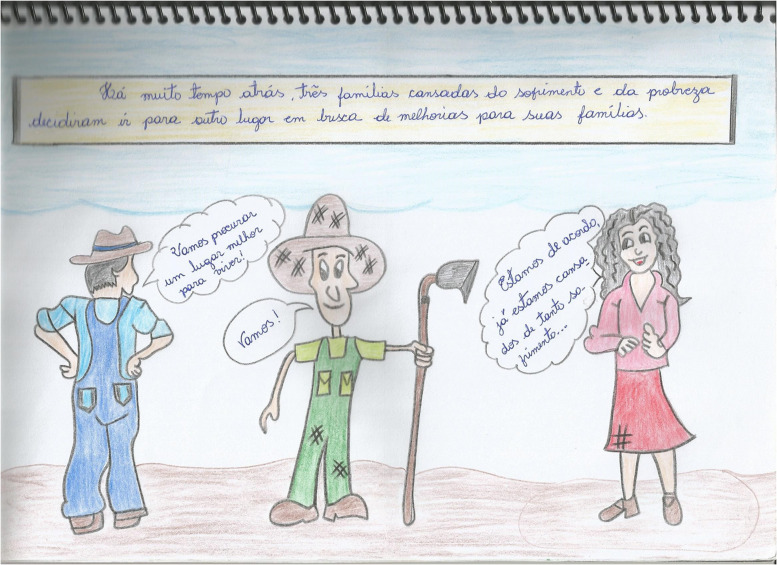
Excerpt from a comic produced by student S1 (source: Elaborated by student S1).

**FIGURE 2:**
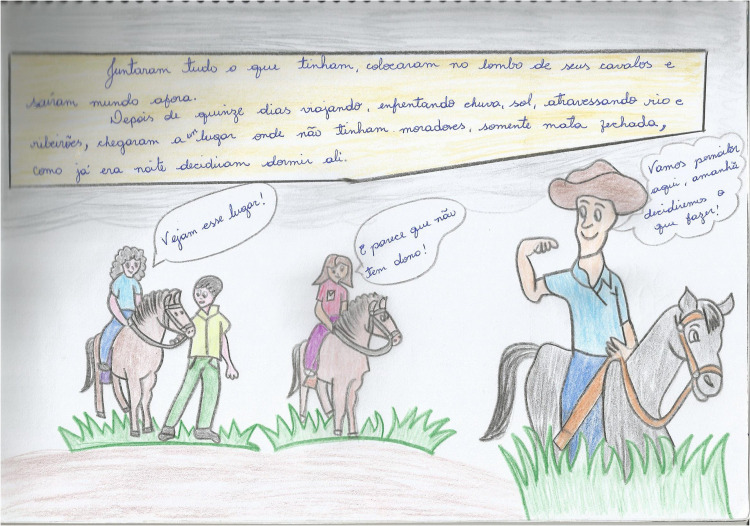
Excerpt from a comic produced by student S1 (source: Elaborated by student S1).

Another story produced by one of the participants of this research revolves around three peasant families who walk day and night in search of useful land to produce sustenance for their people and, from there, to build their lives (Figures 1, 2).

Translated conversation of the drawn history:

Legend: “A long time ago, three families tired of a lot of suffering and poverty decided to go somewhere else, in search of a better life.”

Character on the left: “Let’s find a better place to live!”

Character in the center: “Let’s go!”

Character on the right: “We agree! We are tired of so much suffering!”

Translated conversation of the drawn history:

Legend: “They gathered everything they had, put it on their horses, and walked. After 15 days of traveling, facing sun, rain and crossing rivers, they arrived at a place where there were no residents, only a forest. As it was already night, they decided to sleep there.”

Character on the left: “Look at this place!”

Character in the center: “It seems that nobody lives here!”

Character on the right: “Let’s sleep here! Tomorrow, we decide what to do!”

## Discussion

### Possibilities of Aesthetic Literacy With Young People and Adults via Comic Books

The studies on literacy addressed in this research were important because they helped us characterize aesthetic literacy. In addition, they broadened our understanding of literacy, in line with cultural–historical theory. Our goal was to dialogue with theoretical contributions that support this study, especially from the cultural–historical perspective, because, as Vygotsky (1999) said, concepts should not be unique and immutable but should be constantly revised, as they are working tools that wear out with use.

Writing, Kleiman (1995) noted, is fundamental for people to communicate and socialize with each other. It is necessary to understand and consider other people’s writing—in this case, that of peasant youth and adults. Their life experiences were built in groups with varied literacy practices that, if not in line with the technologized world, dialogue with new ways of understanding reality, of communicating; therefore, of socializing (Stefanello, 2017). Their practices bring with them a world full of cultural objects, experiences, and knowledge, reflected in the arts, in the way they teach, communicate, that is in the way they interact with the world around them. Therefore, literacy studies are important and fundamental to this research.

This reflection allows us to suggest that considering the literacy practices observed in the peasant context is to reinvent the practices of communicating and socializing with the world. Therefore, the school should be dynamic in building a link between what is taught and learned in the field and what is taught and learned in school (Freitas and Rosa, 2015; Street, 2014).

In this direction (Kleiman, 1995, p. 11) emphasizes that the concept of literacy refers to “a set of social practices, whose specific modes of operation have important implications for the ways in which the subjects involved in these practices construct relations of identity and power.” In contributing to the expansion of the concept of literacy (Rojo, 2009, p. 10) states that this term refers to “a very diverse set of situated social practices that involve sign systems, such as writing or other language modalities, to generate meaning.” Adding some ideas to the definition of literacy with youth and adults, Mollica (2009) also states that literacy refers to multiple socio-political-cultural knowledges.

However, Kleiman and Sito (2016) stated that in recent years, the concept of literacy has acquired different designations, due to the varying social practices of reading and writing that have emerged in different cultural contexts and the fact that there is a diversity of verbal and visual languages in society. It is from this moment that multilingualism emerges, which is characterized by the emergence of texts with images. In short, studies about literacy should also address visualities and, therefore, images.

These authors present different ways of conceptualizing literacy but with a similarity: literacy is understood as a social and cultural practice that involves reading, writing, and speaking in different contexts, such as those involving young people and adults. Based on this definition, we can understand aesthetic literacy as something that expands this conception by inserting art into the reading and writing processes of peasant young adult students.

### Assumptions of Cultural–Historical Theory for Developmental Learning

Vygotsky (2009) argues that superior human psychological processes develop from social relationships rather than from dependence on biological factors. For him, the individual assimilates superior psychological functions from the appropriation of culture and develops them in interactions with other people.

Based on this thought, the socio-historical nature of humans defended by the cultural–historical theory and by Vygotsky is fundamental in understanding human rationality and sociability because “it has come to be considered that specifically human skills and characters are not transmitted in a hereditary way, but are adapted through the appropriation of the culture created by previous generations” (Puentes and Longarezi, 2013, p. 249).

In fact, when an individual internalizes an object, it can be socialized through language to other people, for example, artistically, orally, or through written language. However, this is only possible with the development of consciousness and through activities, whereby the individual has created material and immaterial instruments to communicate with the world (Repkin, 2014).

Vygotsky (2009) also argues that good teaching is fundamental to the development of a student’s higher psychic functions. He did not make clear in his studies the understanding of developmental learning—what was to happen with Davídov (1978, 1988, 1997), when elaborating his theory that referred to the study of development learning. This theory originated in the cultural–historical theory from the unfolding of this psychological current highlighted by Vygotsky, mainly concerning the relationship between education and individual development.

Considered one of the most important results of the research developed by Davídov, developmental learning concerns the teacher’s teaching and planning and the development of a student’s psychic functions (Peres and Freitas, 2014; Libâneo, 2016). Thus, one of its main objectives is to promote the development of students’ thinking and autonomy in the performance of tasks and mental actions, which can contribute to their progress in learning (Sforni, 2015).

Davídov’s goal was to propose a theory that would develop theoretical thinking in students. This theory is known as developmental learning. It is important to point out that this learning, with its roots in the cultural–historical theory, is based not only on Vygotsky’s studies but also on Leontiev’s theory of activity and on Marx and Engels’s historical materialism. In fact, in his thesis, Davídov proposes analyzing the movement of reality from the abstract to the concrete to understand how it can be applied in teaching.

In differentiating theoretical from empirical thinking, Davídov (1988) states that in the latter, analysis, abstraction, generalization, and concept are based on external traits; in the former, they are based on the internal traits of the object. In other words, empirical thought is expressed in words, and theoretical thought is expressed in mental actions. According to Kosic (1976), theoretical thought is dialectical and is processed in movement and in constant transformation, unlike empirical thought, which is based on facts arising from experience and systematizing them as absolute truths.

In summary, Davídov (1988) understood that empirical thinking is formed from the comparison of objects and their representations, which makes it possible to separate the most common (general) properties. This general property is known and allows for categorizing certain objects into a given class so that they can be analyzed. At any given moment, it seeks to discover the initial relationship of the integral system with its essence. Thus, empirical thinking (expressed mainly in words) is reflected in the external properties of objects. On the other hand, theoretical thought emerges during the analysis carried out in this integral system; that is, it is mentally elaborated when the objects are transformed from simple representations to mental actions. Thus, theoretical thought (expressed through mental activity) explains the particular and singular manifestations of this system from the general to the particular.

As you can see, teaching focused on the development of theoretical thought causes students to learn to understand an object more broadly, to develop their creativity, to develop as a person, transform the objects around them, and to have autonomy and self-control over their actions. Therefore, from a cultural–historical theoretical perspective, developmental learning can help form an awareness of reality.

Regarding the formation of awareness (Marx and Engels, 1974, p. 20) state that the ideas built by man are directly related to the material activity of men, that is, related to their reality. For this reason, they argue that “it is not awareness that determines life, but it is life that determines awareness,” because being a social product, it will only continue to exist so long as men and their social relations, built along the historical path of humanity, exist.

Consequently, as a manifestation of superior psychological functions and produced by human activity, awareness is a mental activity that relates the individual to reality (Puentes and Longarezi, 2013). In the authors’ view, based on Leontiev and Vygotsky, after the object is internalized by the individual, it can be disseminated to other people through language, making its content socially available.

To broaden this understanding, Freire (1979) describes that in the first moment, reality is not given as a knowable object, since the individual is only trying to have an experience with reality. However, as they become aware of this reality (when they insert themselves into reality with a critical awareness), it is revealed and shows itself in essence to the individual. The author states that awareness does not exist outside of *Praxis*, that is, without the action-reflection act, since it refers to the transformation of the world that characterizes people. Therefore, awareness is historical to the extent that people assume the role of subjects who make and reproduce their reality (essence).

Indeed, “scientific psychology must not ignore the facts of awareness, but materialize them. Without this, any work of teaching, criticism, and investigation is impossible” (Vygotsky, 1999, p. 63). That is, for Vygotsky, there is no word without thought because both are directly related to the process of human mental development; both thought and language are fundamental to understand the nature of human awareness and, consequently, to understand the process of knowledge construction in the individual.

### Participants’ Awareness Based on Verbal Elements of the Comics

The history of student A5 (35 years old) demonstrates the reality of the peasant, which is greatly modified by the interests of capitalism. Many people in the countryside work in agriculture, taking extensive journeys throughout the day to plant and harvest food that reaches people’s tables for their own survival. In addition, land is understood not only as soil but also as a territory and a place to live, work, study, and produce food. It is also understood as a place for conflict. It should not be treated or considered as mere merchandise for agribusiness, which, in trying to make profit and generate capital, destroys countless rivers and vegetation, and pollutes the environment with the justification that it promotes the economic growth of the country with the export of products. With this, we posit the following question: Does elevating and growing the country’s economy imply destroying family farmers’ lands? Does it imply expelling Indians and *quilombolas* from their communities? Does it imply exterminating these populations in conflicts, which are generally violent and increasingly prevalent in the Brazilian countryside? It is the land that peasants build their social and production relations and constitute themselves as historical subjects.

It is also important to emphasize in this analysis the approval by the House of Representatives in 2018 of Bill no. 6.299/2002, known as the “Poison Project,” which advocates greater flexibility in the use of pesticides in plantations in Brazil. In other words, if this bill is sanctioned by the presidency of the republic, there will be a greater amount of poison in the food that goes to the homes of the Brazilian population, which can result in, among other problems, countless diseases caused by these pesticides, such as cancer. There is no doubt that student A5’s story regarding the conflict present in the Brazilian countryside shows another blow from agribusiness suffered by the Brazilian people, especially those who live in the countryside.

In this story produced by the participant in the experiment, once the abstraction of the object is completed, its generalization must occur. For Davídov, “generalization is characterized as a fundamental path for the formation of concepts in students” (1988, p. 59). That is, analysis, abstraction, and generalization are the basis of theoretical thinking. In this sense, the study task becomes the key to study activity (Libâneo, 2016). This activity was only effective after the students completed these tasks.

In other words, after internalizing the concept, the student was able to relate it to different situations of the reality in which this concept is presented in a concrete form. Thus, concrete and abstract are instances of mediation that complement each other dialectically; the abstract improves the understanding of the concrete and the concrete can contribute to advancing the understanding of the abstract. Therefore, the empirical approach typically seen in everyday school action is not sufficient for the formation of theoretical thinking.

From the perspective of the cultural–historical theory, this analysis indicates that during the development of this activity, besides assimilating theoretical knowledge, the student formed consciousness, thus becoming more autonomous and gradually acquiring the ability to learn without the help of the professor, as has occurred in the production of the history reported here.

The formation of this thought during the creation of the stories—going from the general to the particular—made these students’ writing more meaningful by highlighting their emotions, knowledge, lives, and the contradictions present in the rural Brazilian environment. The written stories of these participants in the study are not only an act of communicating with the world around them but also a means of making them present, of exercising their citizenship, and of overcoming the pseudo-concrete reality that ravages peasant territory. Therefore, reading and writing became revolutionary actions for them (Araújo, 2018).

### Aesthetic Literacy in Participants’ Awareness Based on Visual Elements of the Comics

Through this second category of analysis, important aspects of the visual language used to represent the reality of the peasant educator are found in the comic of student S1 (40 years old). The story begins with a catchy title that occupies an entire page: “New Beginning.” It is important to point out that the titles are usually designed with decorative effects that draw the reader’s attention to the story. They are located on the first page of the story and can take different formats, which will vary according to the author’s creativity, just like S1 student did on the first page of her comic book.

Throughout the comic’s pages, the drawings, unlike the other stories, are not in the comics (comic strip contour) because the student deliberately wanted them to occupy all the pages of the story. This means that the comics should not be considered “prisons” that do not allow any element of the scene to escape from its interior. On the contrary, with the student’s creativity and the necessity of the story, it is appropriate and possible for this to happen without negatively affecting the narrative, which was evident in the story she elaborated.

The student conducted the narrative in the comics through the interaction of characters with other elements of the story (scenery, animals, etc.) that she designed herself. In this way, her psychic formations matured sequentially, which made it possible for her to guide the visual reading from the first to the last comic, and from left to right, which led to an understanding of her story.

In this respect, Vygotsky (2009) states that the meaning produced by verbal and visual elements is only possible through the relationship between them in the context of the author’s (student’s) history and life. That is, to understand the essence of a story is to consider, dialectically, the moments from their life in the countryside that enabled them, via thought movement, to have consciousness of their reality, materialized in art.

The countryside for this student is a place of life, work, and prosperity because, by reporting the conquest of the land by the three peasant families, it shows that the social relations produced by them, as well as their knowledge and the needs collectively established by them, were important for them to find a place to live (productive land) and to build their lives.

Based on this reasoning, history shows that the peasant only knows their reality by transforming it and by producing relationships with their social and cultural environment. This reality cannot be understood if the rural peasant is considered an object and not a historical subject (Kosic, 1976). In fact, the reality of the countryside needs to be interpreted in its essence (true) and not in a pseudo-concrete way (appearance/not true).

The student’s story presents an interesting observation: The characters prove to be happy with life in the countryside. This observation is possible based on facial expressions that reveal their emotions. Their faces assume relevance in this process because they allow the reader to question and make inferences about the visual information socialized through the drawings.

From this perspective, it is important to emphasize that the visual elements of these stories have led young people and adults from rural education to produce the gestures and expressions of the characters, evident in the drawings they have created, which leads us to the arguments that images have a high capacity for information, meaning they need to be read and interpreted.

We also point out that the development of the creativity of young people and adults in rural education in the construction of the story designs, as well as the organization of the teaching and study tasks of the experiment, occurred in a different way because they did not work with models, copies, or ready-made concepts characteristic of traditional teaching (Araújo, 2018). That is, the students denied formal logic in the production of their comics.

As can be seen, the conceptual core identified by the student during the activity allowed him to synthesize the studied object, helping him reach theoretical thinking. In this way, the student assimilated and internalized the object through cultural signs, assimilating the general relationship observed in the content worked on. This is fundamental for him to be able to problematize and make inferences about the studied object.

Based on the work of Puentes and Longarezi (2013), we observed that the mastery of the use of material instruments (such as pencils, pens, paper, erasers, rulers, among others), for the elaboration of the drawings of the comics, enabled the student to understand the essence of the phenomenon present in their reality. That is, after developing awareness, he not only identified the visual and verbal signs he used in the stories but also perceived their meanings and thus formed concepts.

This development was only possible through the study activity carried out by them in the experiment because it was during this activity that the young people and adults of rural education developed their mental processes and, consequently, their consciousness as historical subjects in the countryside. In addition, they were able to overcome the difficulties presented throughout the construction of their histories (skills with the drawing, for example), fundamental for advancement in learning.

In this sense, we understand that both words and drawings are important for students to become aware of their reality and are important in forming their higher psychic functions and advancement in aesthetic literacy. By thinking dialectically, the student can understand how the elements relate to the text and how they produce sense and information relevant to their social and cultural environment to overcome the capitalist hegemonic teaching that does not consider their constructed knowledge.

## Significance and Considerations of This Study

Comics are the conceptual core of this research because the students of the rural education system, during the study activity, formed concepts. Thus, they assimilated the general relationship of the content and internalized this object through the verbal and visual signs of the comics. Moreover, the peasant young and adult students were able to create and recreate reality and interpret it through the elements of these unfamiliar drawings in order to develop awareness about the comic book genre. That is, by developing writing and reading through the learning of this art, they developed their psychic functions.

Therefore, students should not be given ready-made concepts and knowledge. On the contrary, one should promote the development of the concept based on their experiences, reality, needs, and through problem situations or questioning. It is necessary for the teacher to propose ways of learning that encourage students to think and conceptualize (Davídov, 1988). Thus, the formation of concepts depends on the performance of tasks present in the study activity, which allow students to exercise their superior psychological functions.

By understanding its nuclear aspect, students develop theoretical thinking and finally arrive at the concept, which enables them to perform tasks with greater autonomy. Therefore, the social and cultural context of these students reflects the true reality of the countryside—deeply marked by social inequalities, low levels of education, conflicts, and the struggle for land.

In the stories analyzed, the participants made clear their actions about the reality of their social and cultural environment, since they announced, through drawings and words, the true reality of their people. In this sense, the students revealed the struggle for an awareness of reality that could be the beginning of the liberating action process. In the words of Freire (1979, p. 21), liberating action is an education that seeks to develop awareness that allows the individual to choose, decide, free himself, and adapt to their environment. In other words, “man cannot participate actively in history, in society, in the transformation of reality, if he is not helping to become aware of reality and of his own capacity to transform it.” Therefore, literacy and awareness are inseparable from the development of the peasant students’ psychic functions, since in the cultural–historical theory, learning is a factor of development and is associated with students’ becoming aware.

Through the research conducted, we understand that aesthetics refer not only to a physical well-made work, but to an object or thought that has more than an artistic value, that is, an object that offers harmony and balance in its forms and therefore, an aesthetic quality. In this context, using artistic language in rural education to help young people and adults to become aware of their reality is a way of thinking about the possibility of multi-literacies, in which aesthetic literacy is an essential aspect.

Therefore, fighting for an education project that is effectively directed toward the peasant population is a way to create new material conditions of life for the men and women of the countryside who, historically, mostly live in conditions of exploitation and submission, as some young people and adults revealed in this study through their comics. In fact, to think that both schools and universities are spaces of intense contradictions, identifying them is essential to transform the reality of the peasants who attend these institutions. This is why the abstractions the participants made in the didactic–formative experiment are important. They managed to capture information about their reality that was unknown to most people.

Therefore, we defend developmental learning in working with young people and adults in rural education because it is a way to promote advancements in teaching and learning through a study activity that enables them to develop superior psychological functions and modify their realities as historical subjects by becoming aware of their own reality through the production of comics.

It is important to emphasize that developmental learning is based on the formation of theoretical thought (psychic activity); therefore, this thought cannot be taught but can only be learned by the student who is a participant in the study activity (Puentes, 2019).

The research suggests that by developing thinking that goes from simple to complex (general to the particular/abstract to the concrete), young people and adults in rural education can also form their awareness of reality, which allows them to have greater autonomy and self-control in the production of comics, as a means of representing and modifying their reality. This study emphasizes that theoretical thinking occurs through a progression from the abstract to the concrete and that this dialectical movement is understood in the totality to which young people and adults in rural education belong.

Furthermore, this study will be useful for the conceptualization and organization of teaching programs, resulting in benefits not only for the academic community but also for students like those involved in this research and for society at large.

## Data Availability Statement

The raw data supporting the conclusions of this article will be made available by the authors, without undue reservation.

## Ethics Statement

The studies involving human participants were reviewed and approved by the São Paulo State University – UNESP. The patients/participants provided their written informed consent to participate in this study.

## Author Contributions

GC, JC, and RG were responsible for designing the research, reviewing the analysis, and writing the manuscript. All authors contributed to the article and approved the submitted version.

## Conflict of Interest

The authors declare that the research was conducted in the absence of any commercial or financial relationships that could be construed as a potential conflict of interest.

## Footnotes

1 Vázquez (1999) argues that the individual needs to have an awareness of a certain object in order to transform it. That is, even if it was not created for aesthetic purposes, but if there is consciousness to create it, if the individual has the ability to create this object, if he has this sensitization, and if the traces of the object’s shape are precise, perfect, and dynamic, it can be considered an object with aesthetic quality.

2 The study activity was based on the formation of concepts that occur in learning (Davídov, 1988). This refers to spontaneous concepts (present before the child goes to school) and scientific concepts (already existing and that join the spontaneous concepts throughout schooling). However, it is necessary that the teacher plans and organizes developmental learning that enables students to perform different tasks and actions so that they can develop these concepts and, therefore, advance in learning and develop their superior psychological functions.
